# Characteristics of cancer patients dying at home during the COVID‐19 pandemic: A study based on vital statistics from 2015 to 2022 in Japan

**DOI:** 10.1002/jgf2.724

**Published:** 2024-08-27

**Authors:** Yu Sun, Rie Masuda, Yuta Taniguchi, Masao Iwagami, Nobuo Sakata, Satoru Yoshie, Jun Komiyama, Kazumasa Yamagishi, Tomomi Kihara, Taeko Watanabe, Hideto Takahashi, Hiroyasu Iso, Nanako Tamiya

**Affiliations:** ^1^ Department of Health Services Research, Institute of Medicine University of Tsukuba Tsukuba Ibaraki Japan; ^2^ Health Services Research and Development Center University of Tsukuba Tsukuba Ibaraki Japan; ^3^ Department of Primary Care and Medical Education, Institute of Medicine University of Tsukuba Tsukuba Ibaraki Japan; ^4^ Department of Health Services Research, Graduate School of Comprehensive Human Sciences University of Tsukuba Tsukuba Ibaraki Japan; ^5^ Institute for Global Health Policy Research Bureau of International Health Cooperation, National Center for Global Health and Medicine Shinjuku‐ku Tokyo Japan; ^6^ Heisei Medical Welfare Group Research Institute Shibuya‐ku Tokyo Japan; ^7^ Department of Home Healthcare Setagaya Memorial Hospital Setagaya‐ku Tokyo Japan; ^8^ Institute of Gerontology University of Tokyo Bunkyo‐ku Tokyo Japan; ^9^ Institute for Future Initiatives University of Tokyo Bunkyo‐ku Tokyo Japan; ^10^ Department of Health Policy and Management, School of Medicine Keio University Shinjuku‐ku Tokyo Japan; ^11^ School of Medicine Hiroshima University Hiroshima City Hiroshima Japan; ^12^ Department of Public Health Medicine, Institute of Medicine University of Tsukuba Tsukuba Ibaraki Japan; ^13^ Department of Public Health, Graduate School of Medicine Juntendo University Bunkyo‐ku Tokyo Japan; ^14^ Faculty of Pharmaceutical Sciences Teikyo Heisei University Nakano‐ku Tokyo Japan

**Keywords:** cancer, COVID‐19, home deaths, visit restrictions

## Abstract

**Background:**

The coronavirus disease 2019 (COVID‐19) pandemic has markedly affected end‐of‐life care, notably increasing home deaths among cancer patients in Japan. This study investigated the characteristics of cancer patients who died at home during the pandemic and the associated factors before and during the pandemic.

**Methods:**

Vital statistics from January 2015 to December 2022 were analyzed to evaluate trends in home deaths among cancer patients aged 0 to 113 years, pre‐pandemic (January 2015 to March 2020) and during the pandemic (April 2020 to December 2022). Home deaths were assessed by demographics, including age, sex, marital status, and residential location. Multivariable modified Poisson regression analyses were performed to identify factors associated with home deaths in both periods.

**Results:**

Among 3,010,374 individuals, 11.6% (226,571/1,959,304) and 20.8% (218,429/1,051,070) died at home before and during the pandemic, respectively. In subgroup analysis depicting the trend of in‐home deaths by patient characteristics, only the age group showed a differential trend: the proportion of in‐home deaths was higher among older people before the pandemic, whereas it was higher among younger people during the pandemic. The multivariable analysis revealed the excess risk of in‐home deaths among people aged ≥65 years before the pandemic and among those aged <65 years during the pandemic.

**Conclusions:**

The pandemic has increased home‐based end‐of‐life care for terminal cancer patients, particularly younger individuals, possibly due to hospital visit restrictions. Ensuring sufficient resources for both home and hospital care is vital to allow individuals to receive end‐of‐life care in their preferred settings.

## Graphical abstract

This research examines the characteristics and trends of home deaths among cancer patients in Japan before and during the COVID‐19 pandemic, using vital statistics from 2015 to 2022. It identifies a significant rise in home deaths among younger patients during the pandemic, likely influenced by hospital visitation restrictions. The study underscores the importance of flexible end‐of‐life care options to accommodate patient preferences.

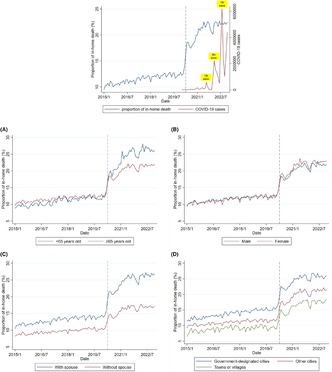

## 1 INTRODUCTION

During the coronavirus disease 2019 (COVID‐19) pandemic, disruptions in healthcare services have been associated with significant changes in the place of death for conditions unrelated to COVID‐19 worldwide, particularly with an increase in home deaths.^1^, ^2^ The rationale for this shift is thought to arise from the pandemic, possibly due to restrictions on hospital visitations and a shortage of beds/resources, coupled with the progress in telemedicine during the pandemic that has enhanced support for patients and caregivers at home.^2^, ^3^, ^4^ Furthermore, certain patients may have died at home because of a lack of timely emergency medical care due to stretched care resources.

In Japan, where the number of deaths per population due to COVID‐19 is relatively low compared with that in many high‐income countries,^5^ there have been reports indicating a heightened proportion of home deaths after the COVID‐19 outbreak, particularly among cancer patients.^6^, ^7^, ^8^ Furthermore, physicians involved in home healthcare observed an increase in both the number of patients passing away at home and the demand for home healthcare services subsequent to the COVID‐19 pandemic, especially among individuals who had cancer, respiratory disease, and dementia.^9^ However, to the best of our knowledge, the distinct attributes of cancer patients that have led to an increased number of home deaths during the pandemic remain unclear.

In meta‐analyses conducted in the pre‐pandemic period, various factors associated with home death among cancer patients have been reported.^10^, ^11^ These factors were categorized into demographic factors (such as age, sex, and area of residence), disease‐related factors (such as type of cancer, time of diagnosis, and functional status), and psychosocial factors (such as extended family support, home care, and its intensity).^10^, ^11^ Previous studies in Japan have also reported that the preferences of patients and their families for home death, along with the availability of home healthcare resources, are associated with home death among cancer patients.^12^, ^13^, ^14^ However, no studies have utilized the nationwide Vital Statistics data, which offers detailed information on actual causes of death, demographic details, and marital status, to explore these associations. Furthermore, changes occurring before and after the COVID‐19 pandemic remain unclear.

To address this knowledge gap, this study aimed to ascertain the patient characteristics that led to increased occurrences of home deaths during the COVID‐19 pandemic, as well as to delineate the factors associated with home deaths both before and during the pandemic. Identifying the characteristics of patients with cancer experiencing home deaths during the COVID‐19 pandemic will enable a more detailed examination of the impact of the pandemic on end‐of‐life care.

## 2 MATERIALS AND METHODS

### 2.1 Study design, data source, and study population

**FIGURE 1:**
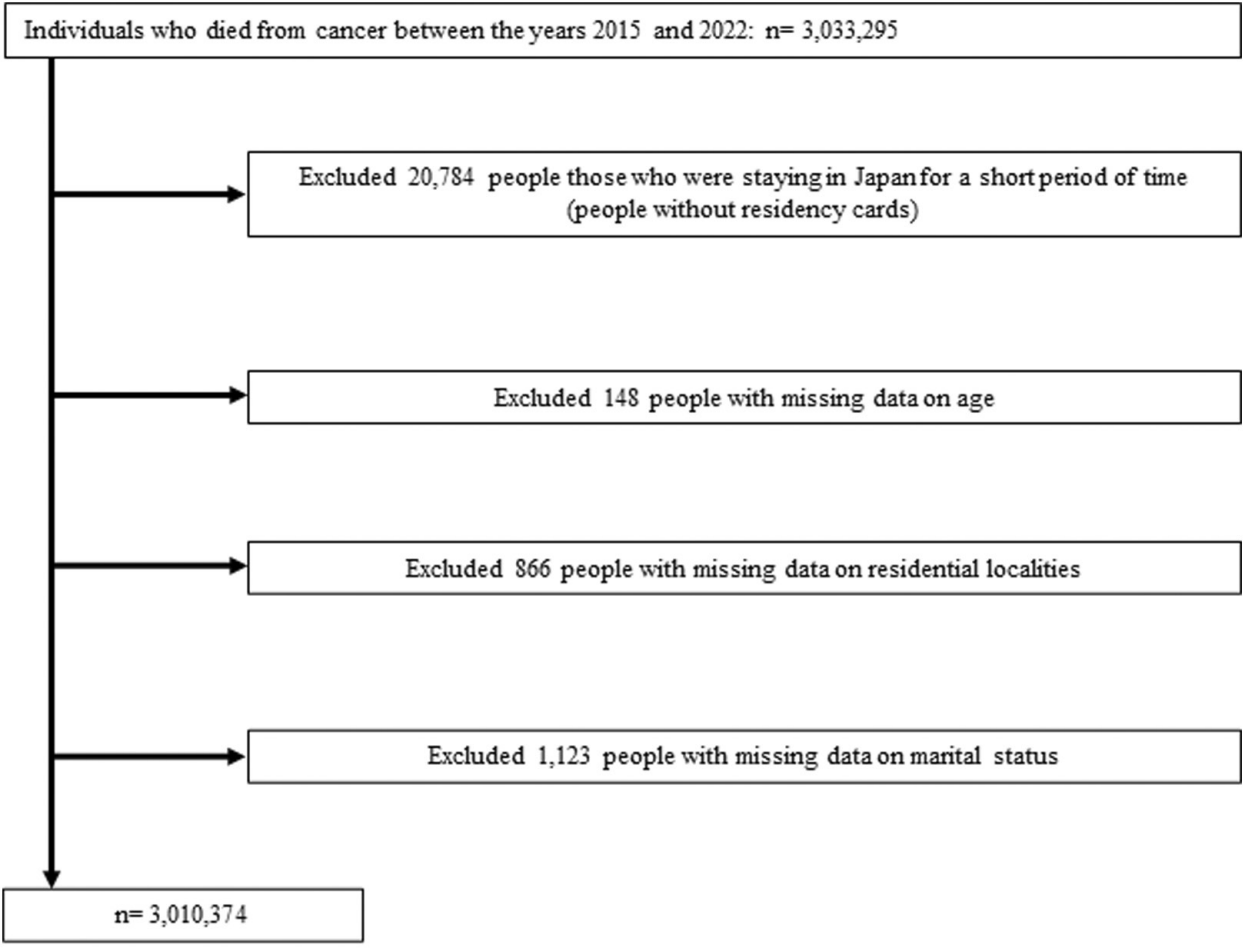
Flow chart of the participant selection.

This study is a retrospective observational study utilizing nationwide vital statistics data from Japan. We acquired the vital statistics on death data in Japan from January 1, 2015, to December 31, 2022, from the Ministry of Health, Labour, and Welfare under the Statistics Act, Article 33. The vital statistics of Japan meticulously record every death, detailing the date and cause, along with the deceased's age, sex, household occupational status, marital status, residential location (both prefecture and municipal classification), and place of death, including hospitals, clinics, nursing homes, older adult care facilities, homes, among all individuals holding residency cards and who have passed away in Japan, irrespective of their nationality.^15^ This study focused on individuals who died of cancer‐related diseases (International Classification of Diseases, 10th revision [ICD‐10] codes C00‐C97), based on the underlying cause of death in the death certificates,^16^, ^17^ which is rigorously maintained under the supervision of the Ministry of Health, Labour, and Welfare. We excluded from the analysis those who were staying in Japan for a short period of time (people without residency cards) and those with missing data on age, residential localities, and marital status (Figure 1).

### 2.2 Outcome and exposure

We divided the observation period into two distinct intervals: pre‐pandemic (January 2015 to March 2020) and during the pandemic (April 2020 to December 2022). We defined the period following April 2020 as the pandemic period because the initial state of emergency was declared on April 7, which resulted in significant alterations to the healthcare system and daily life.^18^ The primary outcome of interest was the incidence of in‐home deaths during each period. The factors examined as potential exposures included age (categorized as <65 or ≥65 years), sex, marital status (with or without a spouse), and residential location, which was stratified based on municipal classification (government‐designated cities or 23 cities within Tokyo, other cities, and towns or villages). We used age as a category to capture potential nonlinear relationships. Government‐designated cities refer to the 20 cities with a population of ≥500,000 people that are designated by the government.^19^ In the categorization of marital status, the “without spouse” category includes individuals who are separated, widowed, or unmarried.

### 2.3 Statistical analysis

First, the monthly in‐home death rate was calculated by dividing the number of in‐home deaths due to cancer by the total number of deaths due to cancer per month from 2015 to 2022. Trends in the percentage of deaths at home for each month are depicted along with the corresponding number of COVID‐19 cases in Japan. Monthly COVID‐19 cases were sourced from the Ministry of Health, Labor, and Welfare website.^20^

Second, we illustrated the trends in in‐home deaths among cancer patients when stratified by age (<65 or ≥65 years), sex, marital status (with or without a spouse), and residential location (government‐designated city or 23 cities in Tokyo, other cities, and towns or villages). The proportion of individuals who died at home among all deceased individuals in each month within each demographic subgroup was calculated and presented graphically. Additionally, the proportion of in‐home deaths during the pre‐ and during‐pandemic periods within each subgroup was determined.

Third, separate univariable and multivariable modified Poisson regression analyses^21^ were conducted for the pre‐ and during‐pandemic periods to identify factors and changes associated with in‐home deaths before and after the onset of the pandemic. Due to changes in outcome frequency between the pre‐ and during COVID‐19 periods, we employed modified Poisson regression, which allowed for direct calculation and comparison of prevalence ratios. This method is preferred over logistic regression analysis, as the latter estimates prevalence ratios from odds ratios, which can be influenced by variations in outcome frequency.^21^, ^22^ In these analyses, the outcome variable was defined as in‐home death, whereas the aforementioned variables (age, sex, marital status, and residential location) served as exposures.

In post‐hoc analysis, for further examination of the difference in age group, we subdivided the categorization of age group (<39, 40–54, 55–64, 65–74, 75–84, ≥85 years) and conducted univariable modified Poisson regression analyses.

All statistical analyses were performed using STATA version 17 (Stata Corp., Texas, USA), with statistical significance set at *p* < 0.05.

## 3 RESULTS

### 3.1 Trends in home death overall

**FIGURE 2:**
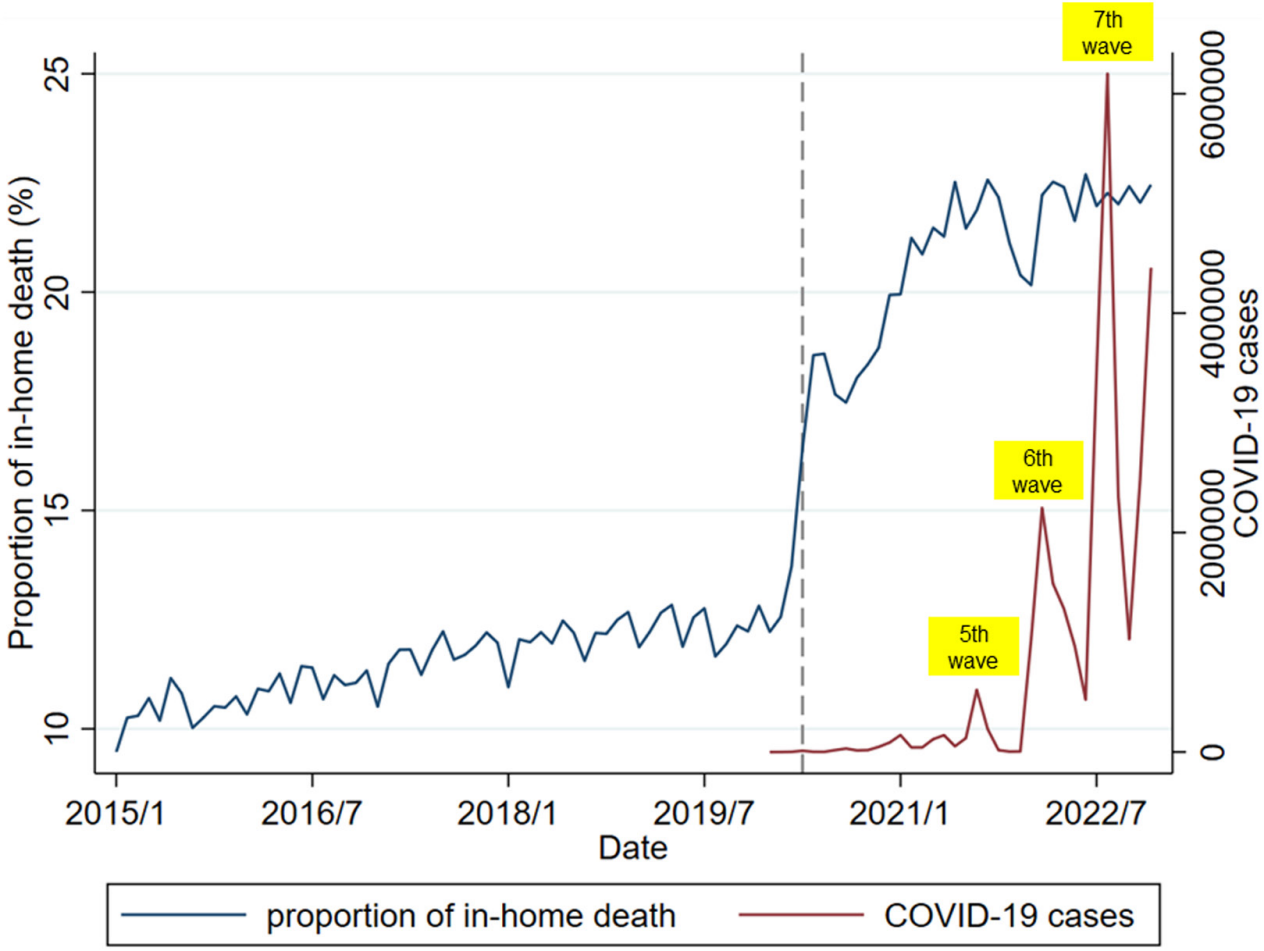
The trends in in‐home deaths among cancer patients and COVID‐19 cases. The gray dashed lines represent the beginning of the COVID‐19 pandemic in Japan (April 2020).

We identified 3,010,374 individuals who died due to cancer between 2015 and 2022 after excluding 22,921 persons with missing data and those without residency cards (Figure 1). Among included individuals, 226,571/1,959,304 (11.6%) died at home in the pre‐pandemic period, and 218,429/1,051,070 (20.8%) died at home in the during‐pandemic period. The monthly trends in in‐home deaths and COVID‐19 cases are shown in Figure 2, which demonstrates an increase in the rate of in‐home deaths following the onset of the COVID‐19 pandemic. Notably, the slight decrease in home death rates from December 2021 to February 2022 coincided with the sixth wave of the pandemic in Japan.

### 3.2 Trends in home death by subgroup

**FIGURE 3:**
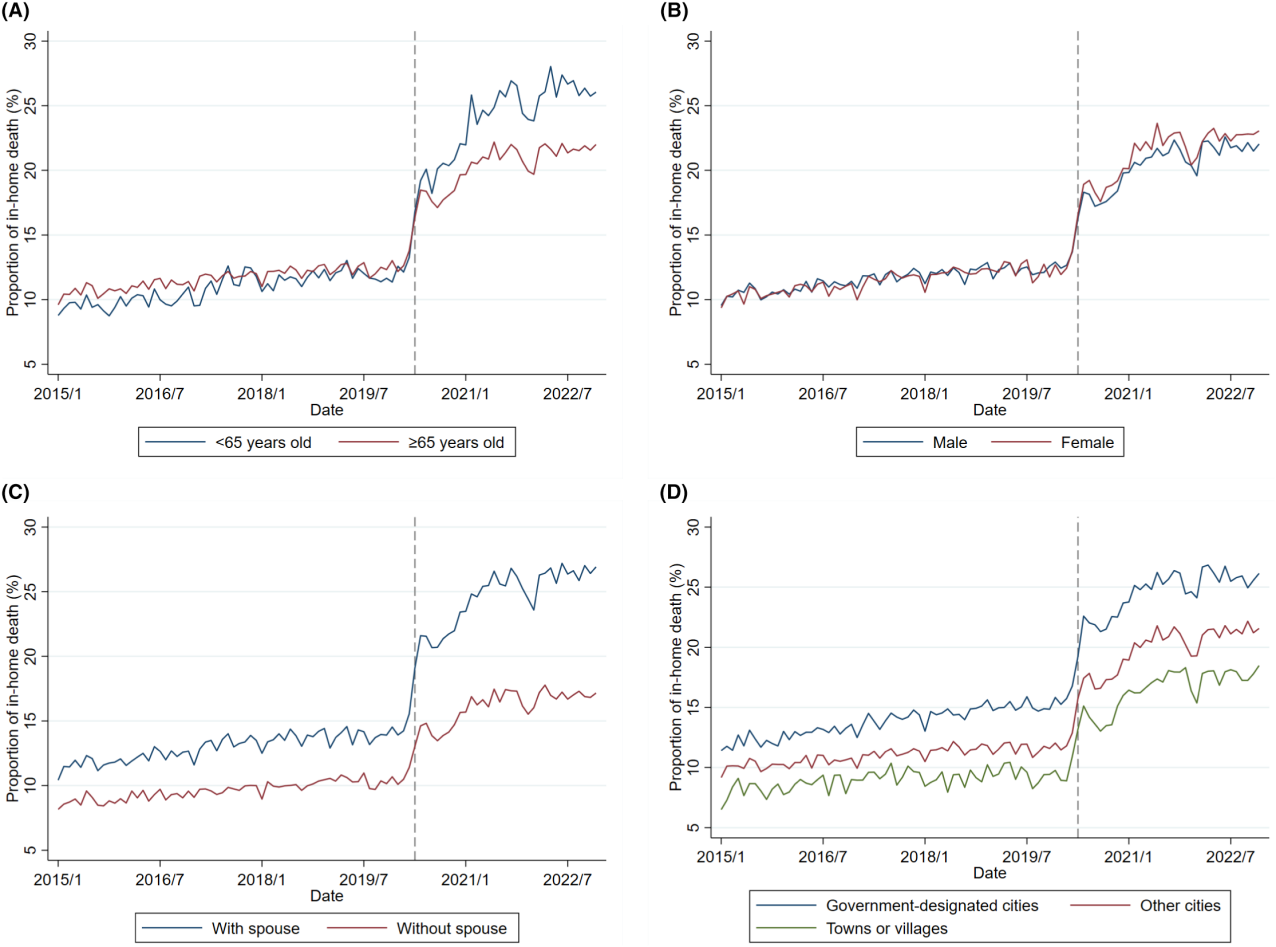
The trends in in‐home deaths among cancer patients in each subgroup. (A) Age, (B) Sex, (C) Marital status, (D) Residential location. The gray dashed lines represent the beginning of the COVID‐19 pandemic in Japan (April 2020). *Designated Cities refers to government‐designated cities or 23 cities within Tokyo.

Figure 3 illustrates the trends in the home death rates for each subgroup. Although the proportion of deaths at home increased across all categories after the onset of the pandemic, only the age group showed significant shifts. Before the pandemic, the proportion of in‐home deaths was higher among those aged ≥65 years than among younger individuals. However, during the pandemic, this shifted, and the proportion of in‐home deaths became higher among those aged <65 years than among older adults. Although the proportion of home deaths was higher among individuals with a spouse than among those without a spouse from the pre‐pandemic period, this difference became more pronounced after the pandemic. Regarding sex, there was a slight increase in home deaths among females than among males during the pandemic. Trends in residential location showed that in‐home deaths were more common in the more populous municipalities from the pre‐pandemic period and increased in all categories during the pandemic period.

Table 1 shows the number and percentage of deaths at home by each patient's characteristics before and during the COVID‐19 pandemic. The proportion of in‐home deaths increased during COVID‐19 in all subgroups. However, the age group with the higher proportion of in‐home deaths shifted from those aged ≥65 to those aged <65: Before the pandemic, the prevalence rates were as follows: <65 years at 10.9% and ≥65 years at 11.7%. During the pandemic, these figures changed to <65 years at 24.0% and ≥65 years at 20.4%.

**TABLE 1:** Characteristics of home death patients due to cancer before and during the COVID‐19 pandemic.

	Pre‐pandemic period^a^ (*n* = 1,959,304)			During‐pandemic period^a^ (*n* = 1,051,070)		
	In‐home death (*n* = 226,571)	Non in‐home death (*n* = 1,732,733)	All (*n* = 1,959,304)	In‐home death (*n* = 218,429)	Non in‐home death (*n* = 832,641)	All (*n* = 1,051,070)
Age category						
<65 years: *n* (%)	29,073 (10.9)	238,625 (89.1)	267,698 (100)	29,649 (24.0)	94,105 (76.0)	123,754 (100)
≥65 years: *n* (%)	197,498 (11.7)	1,494,108 (88.3)	1,691,606 (100)	188,780 (20.4)	738,536 (79.6)	927,316 (100)
Sex						
Male: *n* (%)	133,756 (11.6)	1,018,816 (88.4)	1,152,572 (100)	124,834 (20.4)	486,590 (79.6)	611,424 (100)
Female: *n* (%)	92,815 (11.5)	713,917 (88.5)	806,732 (100)	93,595 (21.3)	346,051 (78.7)	439,646 (100)
Marital status: with spouse						
Without spouse: *n* (%)	81,838 (9.7)	764,946 (90.3)	846,784 (100)	75,977 (16.0)	395,721 (100)	471,698 (100)
With spouse: *n* (%)	144,733 (13.0)	967,787 (87.0)	1,112,520 (100)	142,452 (25.0)	436,920 (100)	579,372 (100)
Type of municipality						
Designated cities^b^: *n* (%)	71,571 (13.9)	444,602 (86.1)	516,173 (100)	68,150 (25.0)	209,541 (75.0)	277,691 (100)
Other cities: *n* (%)	137,311 (11.0)	1,108,525 (89.0)	1,245,836 (100)	133,196 (20.0)	536,598 (80.0)	669,794 (100)
Towns and villages: *n* (%)	17,689 (9.0)	179,606 (91.0)	197,295 (100)	17,083 (16.0)	86,502 (84.0)	103,585 (100)

^a^ Pre‐pandemic period: from January 2015 to March 2020, during‐pandemic period: from April 2020 to December 2022.

^b^ Designated Cities: government‐designated cities or 23 cities within Tokyo.

### 3.3 Factors associated with home death

Table 2 shows the results of univariable and multivariable modified Poisson regression analyses of in‐home deaths before and during the pandemic. In both periods, multivariable analysis showed an increased prevalence ratio for females compared to univariable analysis, with a reversal relationship in the pre‐pandemic period. Notably, individuals aged <65 years exhibited a negative association with home deaths before the pandemic (adjusted prevalence ratio 0.90, 95% CI [0.89–0.91]), whereas this association was positive during the pandemic (1.13 [1.12–1.14]). Such a change did not occur between the pre‐ and during COVID‐19 periods due to factors such as being a female, having a spouse, and residing in a municipality with a larger population, which are associated with in‐home deaths. However, the adjusted prevalence ratio for having a spouse and being female showed a slight increase during the pandemic.

**TABLE 2:** Univariable and multivariable modified Poisson regression analyses of in‐home deaths among cancer patients before and during the COVID‐19 pandemic.

	Pre‐pandemic period^a^ (*n* = 1,959,304)				During‐pandemic period^a^ (*n* = 1,051,070)			
	Univariable		Multivariable		Univariable		Multivariable	
	Prevalence ratio (95% CI)	*p*‐value	Adjusted prevalence ratio (95% CI)	*p*‐value	Prevalence ratio (95% CI)	*p*‐value	Adjusted Prevalence ratio (95% CI)	*p*‐value
Age category								
<65 years	0.93 (0.92–0.94)	<0.001	0.90 (0.89–0.91)	<0.001	1.18 (1.16–1.19)	<0.001	1.13 (1.12–1.14)	<0.001
≥65 years	(reference)		(reference)		(reference)		(reference)	
Sex								
Male	(reference)		(reference)		(reference)		(reference)	
Female	0.99 (0.98–1.00)	0.031	1.11 (1.10–1.12)	<0.001	1.04 (1.03–1.05)	<0.001	1.20 (1.19–1.21)	<0.001
Marital status								
Without spouse	(reference)		(reference)		(reference)		(reference)	
With spouse	1.35 (1.34–1.36)	<0.001	1.40 (1.39–1.41)	<0.001	1.53 (1.51–1.54)	<0.001	1.62 (1.61–1.64)	<0.001
Residential location								
Designated cities^b^	(reference)		(reference)		(reference)		(reference)	
Other cities	0.79 (0.79–0.80)	<0.001	0.79 (0.78–0.79)	<0.001	0.81 (0.80–0.82)	<0.001	0.80 (0.79–0.81)	<0.001
Towns and villages	0.65 (0.64–0.66)	<0.001	0.64 (0.63–0.65)	<0.001	0.67 (0.66–0.68)	<0.001	0.67 (0.66–0.68)	<0.001

^a^ Pre‐pandemic period: from January 2015 to March 2020, During‐pandemic period: from April 2020 to December 2022.

^b^ Designated Cities: government‐designated cities or 23 cities within Tokyo.

The results of the post‐hoc analyses are shown in Appendices S1 and S2. When compared to the age group of ≥85 years, all other age groups, except those <40 years old, showed a negative association with in‐home deaths in the pre‐pandemic period. However, during the pandemic, all age groups exhibited a positive association with in‐home deaths, with the adjusted prevalence ratio increasing as age decreased.

## 4 DISCUSSION

To the best of our knowledge, this is the first study to explore the characteristics of cancer patients experiencing home deaths during the COVID‐19 pandemic using vital statistical data from Japan for the period 2015–2022. Our findings indicate that the proportion of in‐home deaths has risen during the pandemic. Regarding the trends for the proportion of in‐home deaths by patient characteristics, the age group showed a differential change, with the higher proportion of deaths at home shifting from those aged ≥65 years to those aged <65 years around the pandemic. The multivariable modified Poisson regression analyses revealed the excess risk of in‐home deaths among people aged ≥65 years before the pandemic and among those aged <65 years during the pandemic.

This study demonstrated that the proportion of in‐home deaths among cancer patients nearly doubled during the COVID‐19 pandemic. It has been reported that during the pandemic, approximately 30% of palliative care units were partially or completely closed due to their conversion to COVID‐19 dedicated beds or the redeployment of staff.^23^ This suggests that the deficiency of adequate medical resources in hospitals may have contributed to the increase in in‐home deaths. However, the variations in the surge of home deaths across different age groups observed in this study suggest that this increase is rather due to a preference among patients and their families for end‐of‐life care in the home setting, particularly evident among younger patients.

In Japan, the COVID‐19 pandemic prompted widespread visitation restrictions in almost all hospitals to prevent infection from visitors, and 99% of palliative care units have issued visitation restrictions.^23^ Younger patients may have been more likely to receive end‐of‐life care in hospitals before the pandemic, owing to their preference for more aggressive treatments and symptom management, than older patients. However, during the pandemic, there may have been a shift in preference for home‐based end‐of‐life care, driven by the desire to spend meaningful time with loved ones during their final days.^4^, ^9^ A previous meta‐analysis demonstrated varying preferences for in‐home death among younger individuals, with both increased and decreased inclinations observed.^11^ Therefore, the preferences for end‐of‐life care among younger cancer patients may be susceptible to changes in social conditions and available medical resources.

The observation that patients with a spouse exhibited a higher proportion of in‐home deaths even prior to the pandemic is consistent with earlier studies indicating an association between in‐home death and cohabitation with relatives or extended family support.^10^ The observed rise in in‐home deaths among individuals with spouses during the pandemic can also be attributed to the significant impact of visitation restrictions on patients with loved ones, leading to a change in preference for the location of end‐of‐life care.

Females had a higher proportion of in‐home deaths compared to males even before the pandemic, and this slightly increased during the COVID‐19 period. This was consistent with the findings in other countries.^2^ Another previous study reported that females were more likely to discuss their preferred place of death than males.^24^ Therefore, the increased consideration and discussion of end‐of‐life options during the pandemic may have led to a rise in in‐home deaths among females.

Our finding of an increase in home deaths across different residential locations aligns with the results of a prior questionnaire survey conducted among home healthcare providers.^9^ Home deaths were more prevalent in municipalities with larger populations even before the pandemic. Scheduled home‐visit medical care was associated with the proportion of in‐home deaths,^14^ and urban areas have more resources for home medical care.^25^ Therefore, the disparity in resource availability may have influenced these outcomes. With the nationwide increase in home deaths during the pandemic, regardless of municipality size, home care workers in rural areas may have been overburdened. Therefore, during the pandemic, mutual support is required to support home healthcare services, particularly in rural areas with limited healthcare resources.

This study found a slight decrease in deaths at home during the sixth wave of the pandemic, which was consistent across all subgroups. A previous study in the UK also indicated variability in home deaths based on pandemic status.^4^ The sixth wave was characterized by a surge in patients due to an outbreak of the Omicron variant, which potentially made it difficult for medical institutions providing end‐of‐life care at home to adequately respond to preferences for home‐based end‐of‐life care as they were also dealing with a huge surge in fever patients.

This study has some limitations. First, the survey did not include some relevant information, such as economic status and family situations beyond marital status. Incorporating these factors would allow a more detailed identification of the characteristics of cancer patients dying at home before and during the pandemic. Therefore, a more comprehensive survey that includes these factors is desirable. Second, the classification of “death at home” encompassed not only patients' personal residences but also included group homes for older individuals with dementia, home hospice, and older adult housing equipped with care services (*Sabisutsuki koreisya muke jutaku*).^26^ Due to the absence of detailed classifications regarding the place of death in the Vital Statistics, distinguishing between these locations was not possible. However, because our study focused on cancer‐related deaths, we expect minimal impact on the results because the number residing in older adult care facilities, such as group homes, is presumed to be low.

In conclusion, during the COVID‐19 pandemic, we found an increased rate of home deaths among patients with cancer. The age group with the highest proportion of deaths at home shifted from ≥65 to <65 years around the pandemic. Our findings suggest that the pandemic has influenced preferences regarding the location of end‐of‐life care, particularly among younger patients. These findings emphasize the need for tailored end‐of‐life care interventions that consider the unique preferences of different age groups during healthcare disruptions, such as the COVID‐19 pandemic. Additionally, it is crucial to prepare healthcare systems for future crises by ensuring flexibility and adequate resource availability to support end‐of‐life care in both home and hospital settings.

## AUTHOR CONTRIBUTIONS

Y.S. designed the study, analyzed the data, and wrote the initial draft. Y.T. and R.M. contributed to the study design and interpretation and maintenance of the data. N.T. and H.I. contributed to the conception and acquisition of data, study design, interpretation, and revision of the manuscript. All authors approved the final version and are responsible for submitting the manuscript for publication.

## FUNDING INFORMATION

This study was supported by the Japanese Ministry of Health, Labor and Welfare (MHLW) Research Program on Emerging and Re‐emerging Infectious Diseases and Immunization [grant numbers JPMH23HA2011 and JPMH24HA2015]. The funder played no role in the conception, design, implementation, or reporting of this study.

## CONFLICT OF INTEREST STATEMENT

The authors declare that they have no competing interests.

## ETHICS APPROVAL STATEMENT

This study was approved by the Ethics Committee of the University of Tsukuba (approval no. 1754) in accordance with the Declaration of Helsinki. The data are presented in accordance with the STROBE guidelines.

## PATIENT CONSENT STATEMENT

Because we received anonymized data for our analyses, the need for individual participants’ consent was waived.

## CLINICAL TRIAL REGISTRATION

None.

## Supporting information


Appendices S1‐S2

## Data Availability

Data used cannot be shared because the Japanese Ministry of Health, Labor and Welfare owns the original data and only approved its secondary use for the current study. Researchers who meet the criteria may apply directly to the Ministry for permission to use deidentified participant data.
