# Supplementary material for: Characteristics of cancer patients dying at home during the COVID‐19 pandemic: A study based on vital statistics from 2015 to 2022 in Japan
Source: J Gen Fam Med. 2024 Aug 27;25(6):358–65. doi: 10.1002/jgf2.724 (PMC11565075; doi:10.1002/jgf2.724)
Supplement: Supplementary file 1 — Appendices S1‐S2 [file JGF2-25-358-s001.docx]

**Appendix 1.** **Age characteristics of home deaths before and during the COVID-19 pandemic when age categories are subdivided**

|  | Pre-pandemic period* (n=1,959,304)* | | |  | During-pandemic peropd* (n=1,051,070)* | | |
| --- | --- | --- | --- | --- | --- | --- | --- |
|  | In-home death  (n=226,571) | Non in-home death  (n=1,732,733) | All  (n=1,959,304) |  | In-home death  (n=218,429) | Non in-home death  (n=832,641) | All  (n=1,051,070) |
| **Age category:** |  |  |  |  |  |  |  |
| <40 years: n (%) | 1,781 (13.1) | 11,767 (86.9) | 13,548 (100) |  | 1,868 (30.9) | 4,175 (69.1) | 6.043 (100) |
| 40–54 years: n (%) | 8,879 (11.5) | 68,001 (88.5) | 76,880 (100) |  | 9,968 (26.3) | 27,983 (73.7) | 37,951(100) |
| 55–65 years: n (%) | 18,413 (10.4) | 158,857 (89.6) | 177,270 (100) |  | 17,813 (22.3) | 61,947 (77.7) | 79,760 (100) |
| 65–74 years: n (%) | 55,779 (11.5) | 431,254 (88.5) | 487,033 (100) |  | 54,137 (21.8) | 193,711 (78.2) | 247,848 (100) |
| 75–84 years: n (%) | 77,782 (11.7) | 587,004 (88.3) | 664,786 (100) |  | 74,961 (21.1) | 279,762 (78.9) | 354,723 (100) |
| ≥85 years: n (%) | 63,937 (11.8) | 475,850 (88.2) | 539,787 (100) |  | 59,682 (18.4) | 265,063 (81.6) | 324,745 (100) |

*Pre-pandemic period: from January 2015 to March 2020; During-pandemic period: from April 2020 to December 2022

**Appendix 2. Univariable modified Poisson regression analyses of in-home deaths among cancer patients before and during the COVID-19 pandemic when age categories are subdivided**

|  | Pre-pandemic period* (n=1,959,304) | | During-pandemic period* (n=1,051,070) | |
| --- | --- | --- | --- | --- |
|  | Prevalence ratio  (95% CI) | P-value | Prevalence ratio  (95% CI) | P-value |
| **Age category:** |  |  |  |  |
| <40 years | 1.11 (1.06–1.16) | <0.001 | 1.68 (1.62–1.75) | <0.001 |
| 40–54 years | 0.98 (0.95–1.00) | 0.018 | 1.42 (1.40–1.46) | <0.001 |
| 55–65 years | 0.88 (0.86–0.89) | <0.001 | 1.22 (1.20–1.23) | <0.001 |
| 65–74 years | 0.97 (0.96–0.98) | <0.001 | 1.19 (1.18–1.20) | <0.001 |
| 75–84 years | 0.99 (0.98–1.00) | 0.014 | 1.15 (1.14–1.16) | <0.001 |
| ≥85 years | (reference) |  | (reference) |  |

CI: confidence interval

*Pre-pandemic period: from January 2015 to March 2020; During-pandemic period: from April 2020 to December 2022
